# CD4^+^ TSCMs in the Bone Marrow Assist in Maturation of Antibodies against Influenza in Mice

**DOI:** 10.1155/2019/3231696

**Published:** 2019-01-10

**Authors:** Kang Wu, Fei Wang, Guangwu Guo, Yuqing Li, Li-Jun Qiu, Xuefeng Li

**Affiliations:** ^1^Zhongshan School of Medicine, Sun Yat-sen University, Guangzhou 510080, China; ^2^Shenzhen Luohu People's Hospital, The Third Affiliated Hospital of Shenzhen University, Shenzhen 518001, China; ^3^Key Laboratory of Regenerative Biology, Guangdong Provincial Key Laboratory of Stem Cell and Regenerative Medicine, South China Institute for Stem Cell Biology and Regenerative Medicine, Guangzhou Institutes of Biomedicine and Health, Chinese Academy of Sciences, Guangzhou 510530, China; ^4^Department of Neurology, The Central Hospital of Wuhan, Tongji Medical College, Huazhong University of Science and Technology, Wuhan 430014, China

## Abstract

The bone marrow (BM) is not only a reservoir of hematopoietic stem cells but a repository of immunological memory cells. Further characterizing BM-resident memory T cells would be helpful to reveal the complicated relationship between the BM and immunological memory. In this study, we identified CD122^high^ stem cell antigen-1 (Sca-1) ^high^ B cell lymphoma 2 (Bcl-2) ^high^ CD4^+^ stem cell-like memory T cells (TSCMs) as a distinct memory T cell subset, which preferentially reside in the BM, where they respond vigorously to blood-borne antigens. Interestingly, the natural CD4^+^ TSCMs homing to the BM colocalized with VCAM-1^+^ IL-15^+^ IL-7^+^ CXCL-12^+^ stromal cells. Furthermore, compared to spleen-resident CD4^+^ TSCMs, BM-resident TSCMs induced the production of high-affinity antibodies against influenza by B lymphocytes more efficiently. Taken together, these observations indicate that the BM provides an appropriate microenvironment for the survival of CD4^+^ TSCMs, which broadens our knowledge regarding the memory maintenance of antigen-specific CD4^+^ T lymphocytes.

## 1. Introduction

CD8^+^ stem cell-like memory T cells (TSCMs) with properties of self-renewal and multipotency have been well identified in human and mouse [1–3]. Mouse CD8^+^ TSCMs highly express stem cell antigen-1 (Sca-1) and common interleukin- (IL-) 2 and IL-15 receptor beta chain (CD122), as well as B cell lymphoma protein-2 (Bcl-2) at high levels, and human CD8^+^ TSCMs highly expressed CD95 (also called Fas/APO-1) and CD122 [1, 2]. Human CD4^+^ TSCMs were isolated from peripheral blood mononuclear cells of health donors [2]. In particular, human CD4^+^ TSCMs were considered as the HIV-1 latent reservoir [4]. In addition, Th17, as a special CD4^+^ T cell subset-producing inflammation, exhibited a certain degree of stem cell characteristics [5, 6]. However, the existence and function of CD4^+^ TSCMs in mouse, especially at the anatomical site of CD4^+^ TSCMs, were not well characterized.

Previous studies have demonstrated the bone marrow (BM) functions as the major reservoir and site of recruitment for hematopoietic stem cells (HSCs) as well as memory B and T cells by means of providing appropriate niches [7–10]. A common niche that supports HSCs or leukocytes in the BM is constituted by CXCL-12^+^ stromal cells. In certain circumstances, the BM can also support the homeostasis of naïve T cells and pro-B cells [11, 12]. More importantly, BM-resident CD4^+^ T cells show a distinct function from those residing in other organs. For instance, compared with CD4^+^ T cells in the peripheral blood, BM-resident CD4^+^ T cells elicit more efficient activity of inducing the production of high-affinity antibodies. Accordingly, the BM is now regarded as a reservoir of Treg cells to provide an immunosuppressive microenvironment for the maintenance of HSCs [13]. Nevertheless, it has not yet been well characterized whether CD4^+^ TSCMs, as a distinct T cell subset with stem cell property, accumulate in the BM.

Influenza infection may cause serious damage to human health and economy [14]. Antibodies secreted by B cells play an important role in anti-influenza immunity [15]. Many subsets of B cells, including pre-pro-B cells and long-lived plasma cells and memory B cells, preferentially reside in the BM [12]. In mice immunized with T cell-dependent antigen (4-hydroxy-3-nitrophenyl)acetyl-coupled KLH (NP-KLH), the BM-derived CD4^+^ memory T cells could help the maturation of specific antibodies [16]. Of note, human long-lived plasma cells (LLPCs) in the BM were identified to response to the influenza vaccine [17]. Although it is well known that CD4^+^ T cells could help the maturation of antibodies, the relationship between CD4^+^ T cells and B cells in the BM was less characterized.

In this study, we provided evidence that the BM acts as a hub where most of antigen-specific CD4^+^ TSCMs were relocated. Importantly, BM-resident TSCMs showed higher activity in inducing antibodies against influenza when compared with the spleen- (SP-) resident TSCMs in mice. These findings may offer direct implications for immunotherapy against influenza.

## 2. Materials and Methods

### 2.1. Ethics Statement

Animal experiments were carried out following the Sun Yat-sen University Laboratory Animal Center guidelines and were approved by the Institutional Animal Care and Use Committee of Sun Yat-sen University (SYSU-2016-053). Efforts were made to minimize animal suffering.

### 2.2. Mice

OT-II, C57BL/6J, and CD45.1 (B6.SJL-Ptprc^a^Pep3^b^/BoyJ) mice were purchased from Jackson Laboratories and were bred in SPF condition.

### 2.3. Virus and Infection

Infectious influenza A/Puerto Rico/8/34 (PR8) (H1N1) and PR8-OVA were provided by Dr. Zhongfang Wang in Guangzhou Medical University. Viruses were grown in the allantoic cavity of embryonated hen eggs from virus stocks. Lightly anesthetized mice were infected with influenza A virus by intranasal inoculation in 50 *μ*l PBS. The stock of PR8 employed 2 × 10^4^ EID50 (4LD50) and 970 PFU (approximately 2LD50) for PR8-OVA.

### 2.4. Viral Titers

Mice injected with influenza A virus were sacrificed by cervical dislocation at various time points. The lungs were removed, teased into single-cell suspensions in a fixed volume of 5 ml and then 1 ml aliquots frozen, and finally stored at −80°C. The lysates were thawed, and the influenza titers were determined using the Madin-Darby canine kidney (MDCK) cell plaque assay as detailed previously [18, 19].

### 2.5. Flow Cytometry and Sorting

Single-cell suspensions were prepared from the individual mouse spleen, mesenteric lymph nodes, blood, or bone marrow. For staining, cells were preincubated in a 0.1% bovine serum albumin (BSA)/phosphate-buffered saline (PBS) solution of 10 *μ*g/ml anti-FcgRII/III (2.4G2) (eBioscience) for 10 min at 4°C. The cells were then stained for 20 min at 4°C with anti-CD62L (MEL-14), anti-CD45.2 (104), anti-CD44 (IM7), anti-CD3 (145-2C11), anti-Sca-1 (D7), anti-CD8 (53-6.7), anti-IFN-*γ* (XMG1.2), anti-CD4 (RM4-5), anti-BrdU (3D4), anti-CD69 (H1.2F3), anti-CD127 (A7R34), anti-Bcl-2 (3F11), anti-CD183 (CXCR3-173), anti-VCAM-1 (429), anti-CD140b (APB5), and anti-CD31 (MEC13.3). Finally, the cells were stained with live/dead viability (Molecular Probes) to exclude dead cells. For cell sorting, a BD FACSAria II cell sorter (BD Biosciences) was used. For intracellular cytokine staining, cells were stimulated with 100 ng/ml PMA (Sigma) and 1 *μ*g/ml ionomycin (Sigma) in the presence of 5 *μ*g/ml brefeldin A (Sigma) for 4 h. Cells were washed twice in PBS and fixed and permeabilized with BD Cytofix/Cytoperm™ Fixation/Permeabilization Kit. Stained samples were analyzed in a BD LSR II Fortessa (BD Biosciences). Flow cytometric data were analyzed with the FlowJo (Tree Star) software.

### 2.6. Quantitative Real-Time PCR (qRT-PCR)

Total RNA was isolated using TRIzol reagent (Life Technologies) and then subjected to cDNA synthesis with a PrimeScript reverse transcription (RT) reagent kit (Takara). All primers were annealed at 37°C, and RT was processed at 42°C. Quantitative real-time PCR was performed with a SYBR Premix Ex Taq II kit (Takara) following the manufacturer's instructions. The primers in our experiments are listed in Table 1.

### 2.7. *In Vivo* Activation of CD4^+^ T Cells

The 5 × 10^5^ three subsets T cells (naïve T cells: CD3^+^ CD4^+^ CD62L^+^ CD44^−^ Sca-1^−^ CD122^−^; TCMs: CD3^+^ CD4^+^ CD62L^+^ CD44^+^; TSCMs: CD3^+^ CD4^+^ CD62L^+^ CD44^−^ Sca-1^+^ CD122^+^) from the SP or BM of OT-II mice were adoptively transferred to CD45.1 mice. Recipients were immunized with 500 *μ*g of OVA in CFA and sacrificed after 3 days for further analysis.

### 2.8. Frozen Section and Immunofluorescence Staining

Femur samples were fixed in 4% paraformaldehyde (Sigma) for 4 h and equilibrated in 30% sucrose (Sigma)/PBS. After two rinses with cold PBS, samples were decalcified with 0.25 M EDTA (Acros) for 2 days and then embedded in O.C.T. (Sakura). For staining, monoclonal antibodies against VCAM-1 (BD Pharmingen) and CXCL-12 (BD Pharmingen), polyclonal antibodies against IL-7 (Abcam) and IL-15 (Abcam), and cell labeling reagents (Molecular Probes), including 5-(and-6)-carboxyfluorescein diacetate, succinimidyl ester (5(6)-CFDA, SE; CFSE), CellTracker™ Red CMTPX Dye, and DAPI (4′,6-diamidino-2-phenylindole), were used. For secondary antibodies, Alexa Fluor 488 goat anti-rabbit and Alexa Fluor 488 goat anti-rat were purchased from Abcam. Cryostat sections were carried out in Leica CM1900.

### 2.9. Cell Proliferation

Cell proliferation *in vitro* was determined by BrdU. The CD44^low^ CD62L^high^ T cells at 2 × 10^6^/ml from the SP or BM were cultured in RPMI 1640 medium (Gibco) containing 10% FBS (Gibco), penicillin (100 U/ml) (HyClone), and streptomycin (100 *μ*g/ml) (HyClone). For the activation of C57BL/6 J mice-derived T cells, cells were stimulated with anti-CD3 (2 *μ*g/ml) and anti-CD28 (1 *μ*g/ml) (BD Pharmingen) in the presence of IL-2 (10 ng/ml) (PeproTech). For the activation of OT-II mice-derived T cells, experiments were performed by following protocols as described. Briefly, the 1 × 10^6^/ml T cells were cocultured with 2 × 10^7^/ml irradiated T-depleted SP or BM-derived antigen-presenting cells in the presence of OVA_323-339_ peptides *(ISQAVHAAHAEINEAGR)* (2 *μ*M) (AnaSpec) and IL-2 (10 ng/ml) (PeproTech).

### 2.10. BrdU Labeling

For *in vivo* proliferation assay, BrdU (Sigma) was injected at 100 mg/kg with 10 mg/ml in saline intraperitoneally.

### 2.11. Homing Assay

Homing experiments of CMTPX-labeled CD4^+^ TSCMs and CFSE-labeled reference cells were performed as described previously [20]. Briefly, the 2 × 10^6^ CMTPX-labeled (10 *μ*M) CD4^+^ TSCMs were mixed with the same number of newly isolated and CFSE-labeled (10 *μ*M) total spleen cells and then injected intravenously into the CD45.1 recipients. The recipients were sacrificed after 24 h, and cells from the spleen and bone marrow were obtained as described to measure CMTPX^+^/CFSE^+^ ratios by flow cytometry. An aliquot was assessed for the input ratio (IR = (CMTPX)_input_/(CFSE)_input_). The homing index (HI) was calculated as the ratio of (CMTPX)_tissue_/(CFSE)_tissue_ to (CMTPX)_input_/(CFSE)_input_. For instance, a homing index of 1 means that frequency of CMTPX-staining cells was equivalent with that of naïve T cells labeled with CFSE.

### 2.12. Statistical Analysis

Statistical analyses were carried out using the GraphPad 5.0. Data are representative from indicated experiments and are shown as means ± SD. Differences were calculated by one-way ANOVA or *t*-test. ^∗^*P* < 0.05, ^∗∗^*P* < 0.01, and ^∗∗∗^*P* < 0.001.

## 3. Results

### 3.1. CD4^+^ T Memory Stem Cells Preferentially Reside in the BM

**Figure 1:**
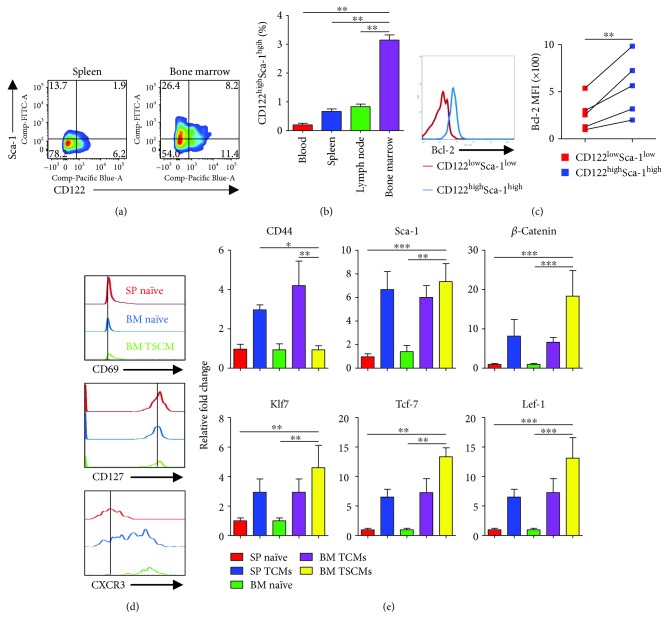
CD4^+^ memory stem cells preferentially reside in BM. (a) Expression of CD122 and Sca-1 in SP- and BM-resident naïve-like T cell compartment. Iridescent cloud represents the frequencies of CD122^high^ Sca-1^high^ subset gated on CD3^+^ CD4^+^ CD8^−^ CD44^low^ CD62L^high^ cells. Data are representative of six independent experiments. (b) TSCMs in various organs. The frequencies of TSCMs accounting for CD44^low^ CD62L^high^ CD4^+^ T cells in the peripheral blood (PB), lymph node (LN), spleen (SP), and bone marrow (BM) were shown as means ± SD, one-way ANOVA. ^∗∗^*P* < 0.01. Data are representative of four independent experiments. (c) Expression of Bcl-2 in naïve and TSCMs subsets in BM-resident T cells. Overlaid histogram plots show the levels of Bcl-2 in BM-resident CD122^low^ Sca-1^low^, CD122^low^ Sca-1^high^, CD122^high^ Sca-1^low^, and CD122^high^ Sca-1^high^ subsets gated on CD3^+^ CD4^+^ CD8^−^ CD44^low^ CD62L^high^ cells. Data are representative of five independent experiments. The MFI of Bcl-2 in CD122^low^ Sca-1^low^, CD122^low^ Sca-1^high^, CD122^high^ Sca-1^low^, and CD122^high^ Sca-1^high^ subsets was shown as means ± SD, one-way ANOVA. ^∗∗^*P* < 0.01. (d) Flow cytometric analysis of BM-resident TSCMs overlaid with SP- and BM-resident naïve T cells. Overlaid histogram lines show expression levels of a given molecule in different CD4^+^ T cell subsets. CD4^+^ T cell subsets were defined as follows: BM-resident TSCMs, CD3^+^ CD4^+^ CD8^−^ CD44^low^ CD62L^high^ CD122^high^ Sca-1^high^; BM- and SP-resident naïve T cells, CD3^+^ CD4^+^ CD8^−^ CD44^low^ CD62L^high^ CD122^low^ Sca-1^low^. Data are representative of five independent experiments. (e) qRT-PCR results show the expressions of *CD44*, *Sca-1*, *β*-catenin, *Klf-7*, *Tcf-7*, and *Lef-1* in different CD4^+^ T cell subsets. CD4^+^ T cell subsets were defined as follows: BM-resident TSCMs, CD3^+^ CD4^+^ CD8^−^ CD44^low^ CD62L^high^ CD122^high^ Sca-1^high^; BM- and SP-resident naïve T cells, CD3^+^ CD4^+^ CD8^−^ CD44^low^ CD62L^high^ CD122^low^ Sca-1^low^. Data are representative of 3 independent experiments (*n* = 3) and shown as means ± SD, one-way ANOVA. ^∗^*P* < 0.05, ^∗∗^*P* < 0.01, and ^∗∗∗^*P* < 0.001.

Although CD8^+^ TSCMs are categorized as memory cells, they display a largely naïve-like phenotype, including CD62L^+^ CCR7^+^ CD45RO^−^ CD45RA^+^ in humans and CD44^low^ CD62L^high^ in mice [21–23]. Given the similarity of CD4^+^ and CD8^+^ T cells on surface markers in most subsets of mice and the consistency of CD4^+^ and CD8^+^ TSCMs surface markers in humans, we sought to apply the criteria of CD8^+^ TSCMs to explore CD4^+^ TSCMs in mice. As expected, significant elevation of the CD122^high^ Sca-1^high^ subset was observed in the BM-derived naïve T cell compartment (Figure 1(a)) when compared with those from other tissues, including the SP, peripheral blood (PB), and mesenteric lymph node (LN) (Figure 1(b)). Thus, we hypothesized that the CD122^high^ Sca-1^high^ TSCMs preferentially reside in the BM.

The well-defined TSCMs have been shown to express not only high levels of CD122 and Sca-1 but also Bcl-2. To validate whether these natural CD122^high^ Sca-1^high^ TSCMs in the BM are completely consistent with the previously well-defined TSCMs, the expression level of Bcl-2 was evaluated in CD122^high^ Sca-1^high^ TSCMs and CD122^low^ Sca-1^low^ naïve T cells. As expected, the expression level of Bcl-2 was higher in CD122^high^ Sca-1^high^ TSCMs than in naïve CD4^+^ T cells (Figure 1(c)), which was consistent with the previously defined TSCMs. Similar to unstimulated naïve T cells, BM-resident TSCMs expressed high levels of CD127 (IL-7R*α*) (Figure 1(d)). Interestingly, the expression of CD69 in BM-resident TSCMs was slightly higher than that of naïve T cells (Figure 1(d)). Surprisingly, similar to CD8^+^ TSCMs in mouse GVHD model, BM-resident CD4^+^ TSCMs also highly expressed CXCR3 molecule (Figure 1(d)). Given the higher expressions of genes that involved in the regulation of stemness of cells in CD8^+^ TSCMs, we also examined these genes referred to a previous report by qRT-PCR (Figure 1(e)). The qRT-PCR data indicated that the mRNA levels of *β*-*catenin*, *Klf7*, *Tcf-7*, and *Lef-1* in BM-resident TSCMs were much higher than those in naïve T cells (Figure 1(e)). It is notable that the mRNA levels of *CD44* and *Sca-1*, as a reference for the efficacy of sorting by flow cytometry, were consistent with the previous report (Figure 1(e)). Collectively, these observations supported that BM-enriched CD122^high^ Sca-1^high^ naïve-like CD4^+^ T lymphocytes can be identified as TSCMs that naturally inhabit the BM.

### 3.2. BM-Resident CD4^+^ TSCMs Vigorously Respond to a Blood-Borne Antigen

**Figure 2:**
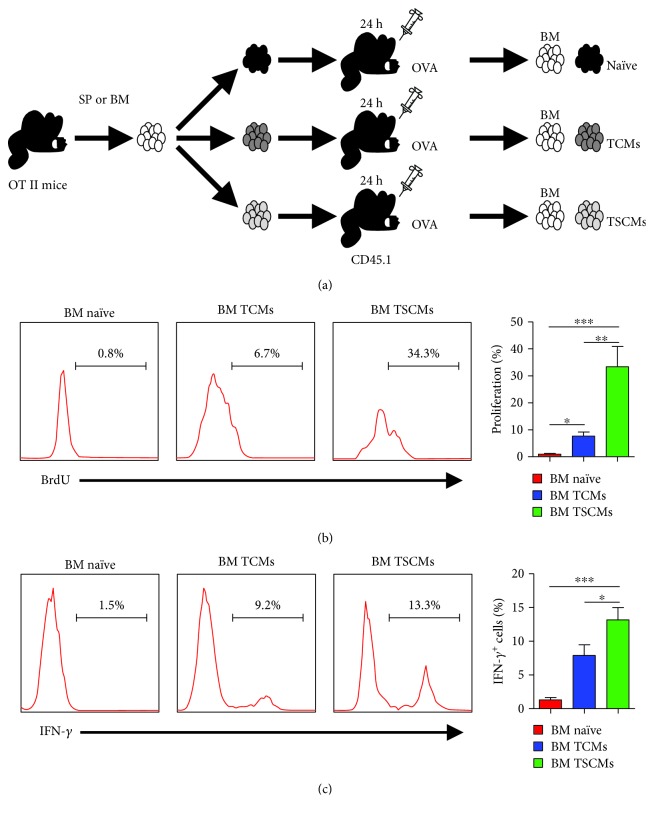
CD4^+^ TSCMs from the BM respond to blood-borne antigens *in vivo*. (a) Schematic diagram of adoptive transfer. (b, c) BM-resident TSCMs possess the capacity of rapidly acquiring effector functions *in vivo*. The 5 × 10^5^ subset of T cells from the BM of OT-II mice was adoptively transferred to CD45.1 mice, respectively. Recipients were immunized with 500 *μ*g OVA in CFA and sacrificed after 3 days for further analysis. The T cell subsets were determined by the following FACS isolations: CD45.2^+^ CD4^+^ CD44^low^ CD62L^high^ CD122^low^ Sca-1^low^ for naïve T cells; CD45.2^+^ CD4^+^ CD44^low^ CD62L^high^ CD122^high^ Sca-1^high^ for TSCMs; CD45.2^+^ CD4^+^ CD44^high^ CD62L^high^ for TCMs. (b) Numbers in histograms represent the percentage of BrdU-positive cells in BM-resident naïve T cells, TCMs and TSCMs after OVA stimulation. (c) Intracellular cytokine staining of naïve T cells, TCMs and TSCMs in the BM. Numbers in histograms show the percentage of IFN-*γ*-expressing cells in the BM after OVA stimulation. Data are representative of three independent experiments (*n* = 6). Frequencies of BrdU^+^ (b) and IFN-*γ*^+^ cells (c) were shown as means ± SD, *t*-test. ^∗^*P* < 0.05, ^∗∗^*P* < 0.01, and ^∗∗∗^*P* < 0.001.

As previously reported, CD4^+^ TSCMs can elicit rapid immune response upon antigen rechallenge [23]. To investigate the immune response of CD4^+^ TSCMs in situ, purified naïve T cells (CD4^+^ CD44^low^ CD62L^high^ CD122^low^ Sca-1^low^) and central memory T cells (TCMs) (CD4^+^ CD44^high^ CD62L^high^) and TSCMs (CD4^+^ CD44^low^ CD62L^high^ CD122^high^ Sca-1^high^) from the BM of OT-II mice (CD45.2^+^) were adoptively transferred into congenic mice (CD45.1^+^), followed by antigen stimulation using ovalbumin (OVA) immunization (Figure 2(a)). Flow cytometric analysis showed that BM TSCMs displayed much higher levels of cell proliferation and IFN-*γ* production when compared with those of BM TCMs and BM naïve T cells (Figures 2(b) and 2(c)). These results indicated that BM-enriched CD122^high^ Sca-1^high^ TSCMs respond to a blood-borne antigen efficiently.

### 3.3. Preferential Migration of CD4^+^ TSCMs to the BM

**Figure 3:**
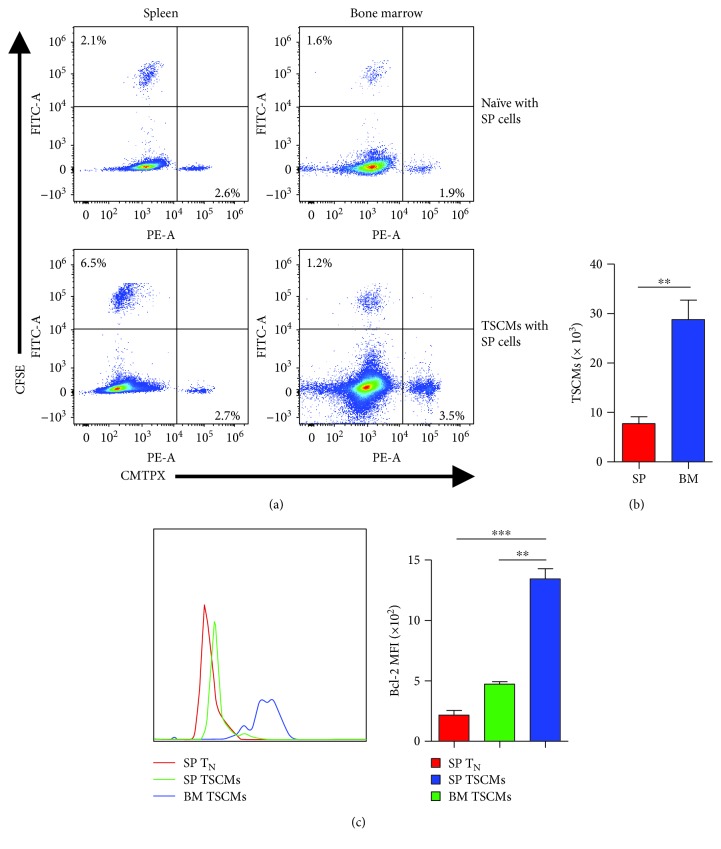
Homing of TSCMs to the BM. (a) Comparison of the homing index of naïve T cells and CD4^+^ TSCMs. Data are representative of three independent experiments. (b) Numbers of transferred TSCMs in the SP and BM. Data were representative of three independent experiments (*n* = 6). The numbers of CD45.2^+^ CD4^+^ CD44^low^ CD62L^high^ CD122^high^ Sca-1^high^ cells were shown as means ± SD, *t*-test. ^∗∗^*P* < 0.01, ^∗∗∗^*P* < 0.001. (c) High expression of Bcl-2 in CD4^+^ TSCMs relocated in the BM. The 2 × 10^6^ CD44^low^ CD62L^high^ CD4^+^ T cells sorted from the spleen or bone marrow in C57B6/J mice were adoptively transferred to CD45.1 mice. The overlaid histogram shows the expressions of Bcl-2 of CMTPX-positive cells in BM and SP cells. Data are representative from three independent experiments and shown as means ± SD (*n* = 6), one-way ANOVA. ^∗∗^*P* < 0.01, ^∗∗∗^*P* < 0.001.

Different from CD8^+^ TSCMs, it was not impacted that the generation of CD4^+^ TSCMs was dependent on *β*-catenin signaling pathway. It is indispensable to purify the CD4^+^ TSCMs from the BM to perform the homing assay. The purified CD4^+^ TSCMs were labeled with CMTPX and then mixed with CFSE-labeled total SP cells (as reference) at ratio of 1 : 1. The mixed cells were injected into recipient mice. After 12 hours, we detected the ratios of CMTPX-positive cells and CFSE-positive cells and then calculated the homing index. Obviously, the homing index of CD4^+^ TSCMs in the BM was higher than that in the SP (Figures 3(a) and 3(b)). In addition, we also compared the protein levels of Bcl-2 in SP- and BM-resident CD4^+^ TSCMs to validate their phenotypes. Importantly, the expression of Bcl-2 in BM-resident CD4^+^ TSCMs was much higher than that of SP-resident CD4^+^ TSCMs (Figure 3(c)). Taken together, these data demonstrated that the CD122^high^ Sca-1^high^ TSCMs preferentially homed to the BM.

### 3.4. CD4^+^ TSCMs Are Attached to VCAM-1^+^ IL-15- or IL-7-Expressing Stromal Cells in the BM

**Figure 4:**
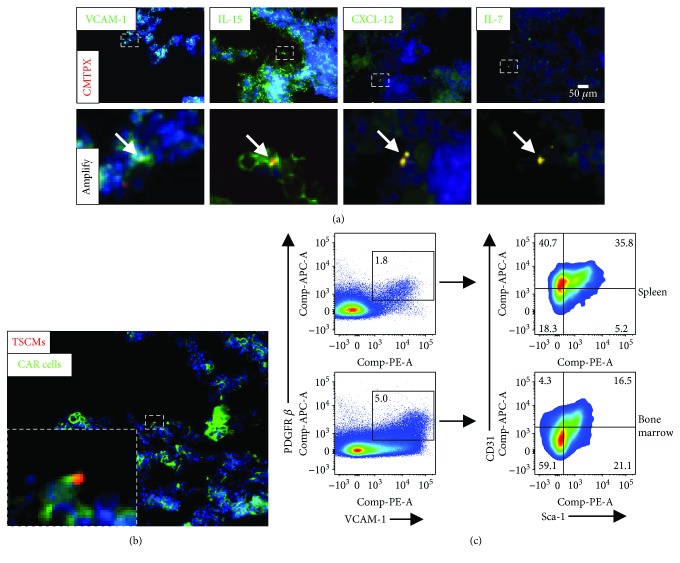
CD4^+^ TSCMs attach to VCAM-1^+^ IL-15- and IL-7-expressing stromal cells. (a) Colocalizations of CD4^+^ TSCMs with VCAM-1^+^ IL-15- and IL-7-expressing stromal cells. The 1 × 10^6^ CD4^+^ TSCMs isolated from the BM were labeled with CMTPX (red) and then injected to CD45.1 mice. After 24 h, the recipients were sacrificed and bone tissues were conducted for immunofluorescence staining. The immunofluorescence images show a representative picture of anti-VCAM-1 (green), IL-15 (green), CXCL-12 (green), or IL-7 (green) versus CMTPX-labeled TSCMs (red). The scale bars indicate that the actual distances on the specimen are 50 *μ*m. Data are representative of three independent experiments. (b) Colocalizations of TSCMs with VCAM-1^+^ PDGFR*β*^+^ CD31^−^ Sca-1^−^ cells. The 5 × 10^5^ TSCMs sorted from the C57B6/J mice BM were labeled with CMTPX (red, 5 *μ*M) and mixed with 1 × 10^6^ VCAM-1^+^ PDGFR*β*^+^ CD31^−^ Sca-1^−^ cells (from the BM) labeled with CFSE (green, 10 *μ*M). Cells were injected into CD45.1 mice. After 24 h, the recipients were sacrificed and conducted frozen sections. The photo shows a representative picture of CFSE-staining VCAM-1^+^ PDGFR*β*^+^ CD31^−^ Sca-1^−^ cells (CXCL-12-abundant reticular cells, CAR cells) versus CMTPX-labeled TSCMs. The scale bar indicates that the actual distance on the specimen is *50 μ*m. (c) Flow cytometric analysis of VCAM-1^+^ PDGFR*β*^+^ CD31^−^ Sca-1^−^ cells in the SP and BM. Dot plots represent the frequencies of VCAM-1^+^ PDGFR*β*^+^ cells (left) and CD31^−^ Sca-1^−^ cells (right) in the SP and BM. Data are representative for three independent experiments (*n* = 5). Frequencies of VCAM-1^+^ PDGFR*β*^+^ CD31^−^ Sca-1^−^ cells were shown as means ± SD, *t*-test. ^∗^*P* < 0.05, ^∗∗^*P* < 0.01, and ^∗∗∗^*P* < 0.001.

CD122 (IL-2R*β*) is also shared as the IL*-*15 receptor, and the persistence of TSCMs is dependent on IL-7 and IL-15, whereas IL-15 is highly expressed in stromal cells in the BM, which overlap with IL-7-expressing cells. Thus, we speculated that these cells could constitute the appropriate niches for BM-resident TSCMs [16, 24, 25]. To validate this hypothesis, the colocalization of TSCMs with stromal cells in the BM was examined by cryofluorescence. TSCMs were clearly colocalized with VCAM-1^+^ IL-7- or IL-15-expressing stromal cells (Figure 4(a)). In addition, TSCMs were also located adjacent to CXCL-12^+^ stromal cells (Figure 4(a)). According to the previous report, the IL-7- and IL-15-expressing cells were constituted by CXCL-12-abundant reticular (CAR) cells whose surface marker is VCAM-1^+^ PDGFR*β*^+^ CD31^−^ Sca-1^−^. It is indispensable to investigate whether BM-resident CD4^+^ TSCMs are colocalized with CAR cells. To test our hypothesis, the VCAM-1^+^ PDGFR*β*^+^ CD31^−^ Sca-1^−^ cells were isolated from the BM and transferred into recipient mice with BM CD4^+^ TSCMs. As expected, the cryofluorescence results represent that CD4^+^ TSCMs were surrounded by CAR cells in the BM (Figure 4(b)). Further flow cytometric analysis confirmed the significantly higher levels of VCAM-1^+^ PDGFR*β*^+^ CD31^−^ Sca-1^−^ stromal cells in the BM (Figure 4(c)). These observations indicated that TSCMs are attached to CXCL-12^+^ VCAM-1^+^ IL-7- and IL-15-expressing stromal cells in the BM.

### 3.5. BM CD4^+^ TSCMs Assist in Affinity Maturation of Antibodies against Influenza In Vivo

**Figure 5:**
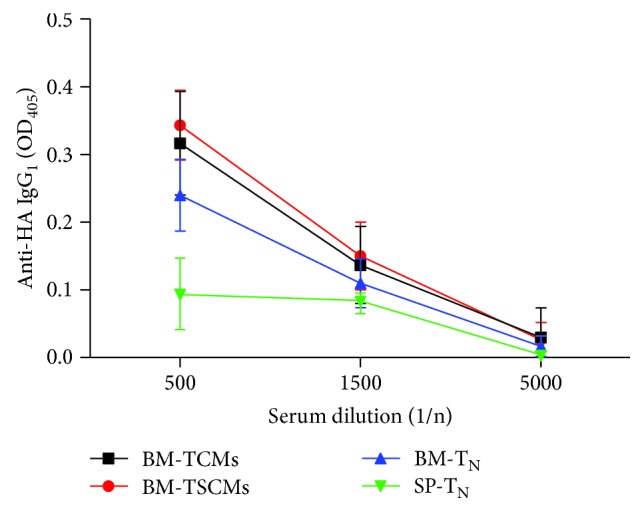
BM memory CD4^+^ TSCMs assist in affinity maturation of antibodies *in vivo*. Transferred CD4^+^ TSCMs from the BM provide help for affinity maturation of antibodies *in vivo*. 4 × 10^6^ CD19^+^ CD138^−^ CD4^−^ cells (naïve B cells) from OT-II mice SP were transferred with 1 × 10^5^ CD4^+^ CD44^low^ CD62L^high^ CD122^high^ Sca-1^high^ T cells from the BM or SP of OT-II mice, into CD45.1 mice. One day later, recipients were immunized with 2000 hemagglutination units PR8-OVA in CFA. The blood taken from transferred mice 5 days later was analyzed for anti-HA IgG_1_ by ELISA. Data are representative of three independent experiments, means ± SD. *n* = 6.

CD4 T cells, also known as helper T cells, assist CD8 T cell-mediated cellular immunity and B cell-mediated humoral immunity in antivirus infection. To explore the relationship between BM CD4^+^ TSCMs and BM B cells, naïve B cells were transferred together with BM CD4^+^ TSCMs and other T cell subsets (including naïve T cells and memory T cells) from the BM or SP of unimmunized mice into recipient mice which were then immunized with influenza A virus. Apparently, recipient mice with transferred BM CD4^+^ TSCMs secreted more influenza antibodies in serum (Figure 5). These data demonstrated that BM CD4^+^ TSCMs provide help for affinity maturation of antibodies against influenza *in vivo*.

## 4. Discussion

The BM is known to play an important role in controlling immune responses by influencing the generation of lymphocytes and the maintenance of immunological memory [16, 26–30]. Although CD4^+^ TSCMs could relocate in the BM, the unambiguous anatomical sites of transition from the naïve state have not yet been determined. Of note, the specific adhesion molecules involved in the process of the relocation of CD4^+^ TSCMs in the BM were still not determined. In this study, CD4^+^ TSCMs that highly expressed CD122, Sca-1, and Bcl-2 were found to reside in the BM-resident naïve-like T cell compartments unambiguously. Although a small number of natural TSCMs were detected in the peripheral lymphoid organs, including the SP, PB, and LN, the frequencies of natural TSCMs in these organs were much lower than those in the BM. Notably, similar to SP-derived TSCMs, the BM-resident TSCMs were capable of acquiring effector functions more rapidly upon blood-borne antigen exposure when compared with naïve T cells. In fact, these data also suggest the significant accumulation of TSCMs in the BM rather than being simply confined to peripheral inflammatory sites. Despite the concomitant expression of numerous markers of naïve T cells on the surface of TSCMs, both bioinformatic analysis of microarray data and antigenic stimulation experiments have suggested that TSCMs are most closely related to TCMs [22, 31]. However, it remains unknown whether the other characteristics of TSCMs, especially the trafficking properties, are also similar to those of TCMs. Through the calculation of homing index of CD4^+^ TSCMs, we found the preferential relocation of CD4^+^ TSCMs in the BM.

An appropriate microenvironment for BM-resident TSCMs requires an architecture facilitating homeostasis of TSCMs in specific areas. These conditions seem to be met in BM stroma, which consists of a fibrous network of VCAM-1^+^ IL-7- and IL-15-expressing stromal cells within VCAM-1^+^ PDGFR*β*^+^ CD31^−^ Sca-1^−^ stromal cell compartments [24, 25]. Consistent with the previous report, the expression level of CD127 molecule was downregulated in BM CD4^+^ TSCMs by IL-15 signaling from stromal cells [32, 33]. In particular, the niches in the BM could provide not only signaling of homeostatic proliferation (provided by IL-15 signaling) but also a survival signal (provided by the pair of IL-7 and CD127) through upregulating the expression of Bcl-2 for the maintenance of TSCMs. The cooperation of these two signaling pathways (IL-7 and IL-15 signaling) supported the residence of CD4^+^ TSCMs in the bone marrow and the recalling of CD4^+^ TSCMs in the influenza model. In contrast, we could not detect the colocalization of TSCMs with conventional APCs, including B220^+^, CD11C^+^, and F4/80^+^ cells (data not shown). Strikingly, although the result of cell cycle indicated that BM-resident TSCMs were in resting status, the expression of CD69 in BM-resident TSCMs was slightly higher than that of naïve T cells. Simultaneously, BM-resident TSCMs could be reactivated rapidly upon exogenous antigen invasion, which provided a hint that active TSCMs are more likely to reside in areas close to APCs. We speculated that the antigen-specific responses might be accompanied by the confluence of TSCMs to large aggregates with several APCs, leading to the activation of TSCMs in the BM. Given that a minority of TSCMs was found in the periphery immune organs and a large number of TSCMs rapidly accumulated in inflammatory sites, we propose that these cells utilize the niches in the BM as a refuge and can be temporarily hidden from antigenic exposure before executing immunological surveillance. Of note, the CAR cells surrounded the CD4^+^ TSCMs in the BM and the frequency of CAR cells in the BM was much higher than that in the SP, which might be a key factor affecting the dynamics of blood-borne TSCM migration *in vivo*.

The severity of influenza infection depends mainly on the type of influenza virus and the status of the patient's immune system [34], since cancer patients who receive bone marrow or stem cell transplants have decreased immune and poor responses to influenza vaccines [35, 36]. In our study, we have demonstrated that BM CD4^+^ TSCMs that could help the maturation of antibodies against influenza were very important for anti-influenza immune. In the clinical bone marrow transplantation, T cells, including CD4^+^ T cells and CD8^+^ T cells, must be depleted from donor's BM cells to reduce graft-versus-host disease [37–39]. Perhaps, with the transplantation of a small amount of CD4^+^ TSCMs from the donor BM, the anti-influenza immune of transplant recipients can be effectively improved to resist infection after BM transplantation. Although our data have demonstrated that the BM CD4^+^ TSCMs could help the maturation of antibodies against influenza produced by B cells, it remained unknown whether BM CD4^+^ TSCMs could help B cell-secreted antibodies against other fatal diseases.

Our data indicate that the BM-resident TSCMs exert much stronger activity of inducing production of antibodies against influenza, which could be instructive for development of anti-infection immunotherapy [40]. In melanoma patients, high frequencies of tumor-specific T cells could be detected by HLA tetramer binding; nevertheless, most of these cells were anergic or nonresponsive [41]. Compared with other subsets, TSCMs show higher immune activity. Although the expression of IFN-*γ* and cell proliferation of natural SP-derived TSCMs was almost equal to those of natural BM-derived TSCMs, we exploited the fact that the microenvironment in the BM could generate more functional CD4^+^ TSCMs *in vivo*. Our data suggest that the selection of a proper microenvironment for functional CD4^+^ TSCMs might be a novel direction to improve the efficacy of immunotherapies.

Overall, our study has revealed that TSCMs, as a distinct member of the memory cell club, exist naturally in wild-type mice and principally accumulate in the BM. In addition, these findings have revealed the relationship between BM CD4^+^ TSCMs and anti-influenza immunity. It is intriguing that the BM might function as a type of “nest” for long-lived cells, including HSCs, conventional memory B and T cells, and TSCMs, by providing dedicated and distinct niches to each of them [42–44]. Moreover, these findings open the door to the development of a new infection disease immunotherapy strategy by driving the pathogen-specific TSCMs out from the BM of patients who suffer from malignant viral infections.

## Figures and Tables

**Table 1:** Primers used in this study.

*CD44* forward	AAAAAGCCATGCAGCAGCTC
*CD44* reverse	TTGCCTCTTGGGTGGTGTTT
*Sca-1* forward	CCACATCTGACAGAACTTGCC
*Sca-1* reverse	GCTGCACAGATAAAACCTAGCAG
*β*-*Catenin* forward	CGCCGCTTATAAATCGCTCC
*β*-*Catenin* reverse	TTCACAGGACACGAGCTGAC
*Klf7* forward	CGTTGAAACTGGTGGCCAAG
*Klf7* reverse	ATAAACTTTCCGGCACCCGT
*Tcf 7* forward	GTACATGGAGAAGCCGAGGG
*Tcf 7* reverse	ACTCTGGAAGTTTGTCCGGG
*Lef-1* forward	AGCACGGAAAGAGAGACAGC
*Lef-1* reverse	GCTGTCATTCTGGGACCTGT
*GAPDH* forward	GGACCTCATGGCCTACATGG
*GAPDH* reverse	TAGGGCCTCTCTTGCTCAGT

## Data Availability

The data used to support the findings of this study are available from the corresponding author upon request.
